# Exploratory characterization of dynamic soluble programmed death-ligand 1 trajectories and their association with mortality in critical coronavirus disease 2019

**DOI:** 10.1186/s40560-025-00846-3

**Published:** 2026-01-05

**Authors:** Shungo Takeuchi, Eiji Kawamoto, Takashi Matsusaki, Daisuke Ono, Yosuke Sakakura, Arong Gaowa, Eun Jeong Park, Motomu Shimaoka, Ryuji Kaku

**Affiliations:** 1 Department of Anesthesiology, Mie University Graduate School of Medicine, Mie University, Tsu, Japan; 2 Department of Molecular Pathobiology and Cell Adhesion Biology, Mie University Graduate School of Medicine, Mie University, Tsu, Japan

**Keywords:** COVID-19, Soluble PD-L1, Immune checkpoints, Organ dysfunction, ICU mortality, Machine learning

## Abstract

**Background:**

Persistent immune checkpoint activation is a recognized feature of critical coronavirus disease 2019 (COVID-19). However, the temporal behavior and clinical utility of soluble programmed death-ligand 1 (sPD-L1) remain unclear. We investigated the longitudinal changes in sPD-L1, its relationship with organ dysfunction markers, and their prognostic value when combined with machine learning (ML) models.

**Methods:**

In this single-center observational study, we included 40 adults with severe COVID-19 pneumonia admitted to the intensive care unit (ICU) (April 2021–December 2022), and 23 healthy volunteers as controls. We measured plasma sPD-L1 on ICU day 1, 5, 7, 14, and 21. Routine biochemistry, complete blood counts, and arterial blood gas analyses were conducted in parallel. Cox regression was used to identify independent predictors of hospital mortality, the primary outcome. Eight ML classifiers were trained using admission variables and sPD-L1 levels from ICU day 1, 5, and 7. Discrimination was assessed using stratified fivefold cross-validation, and feature importance was evaluated using Shapley Additive Explanations (SHAP).

**Results:**

Of 40 patients, 10 died during hospitalization. Overall, sPD-L1 levels declined during the ICU stay but remained persistently high in non-survivors. ICU day 5 and 7 values differed significantly between survivors and non-survivors (*p* = 0.023 and 0.001, respectively). In multivariable Cox analysis, ICU day 7 sPD-L1 levels and arterial lactate levels on admission independently predicted mortality. ICU day 7 sPD-L1 levels correlated positively with creatinine, C-reactive protein, and fibrinogen levels (all *p* < 0.05) in cross-sectional correlation analyses. Among ML models, the support vector machine achieved the highest discriminative accuracy (mean area under the curve = 0.917). ICU day 5 sPD-L1 was designated as the primary predictor of mortality based on SHAP analysis, with lactate contributing minimally.

**Conclusion:**

Sustained sPD-L1 elevation during the first ICU week is strongly associated with early organ dysfunction and independently predicts death in critical COVID-19. Incorporating serial sPD-L1 measurements into bedside ML models significantly enhances risk discrimination. These findings support sPD-L1 as an integrative biomarker of the immune–renal–coagulation interplay, warranting validation in larger multicenter cohorts and exploration as a potential companion marker for immune-modulatory interventions.

**Supplementary Information:**

The online version contains supplementary material available at 10.1186/s40560-025-00846-3.

## Background

Severe coronavirus disease 2019 (COVID-19) requiring intensive care is associated with high mortality, with intensive care unit (ICU) mortality rates for patients with COVID-19 who are critically ill averaging 30–40% across various studies and settings [1]. An early “cytokine storm” hyperinflammatory phase of COVID-19 has been well recognized [2, 3]; however, there is growing evidence that many patients with critical illness also develop immune exhaustion or secondary immunosuppression [4]. Marked lymphopenia and T-cell dysfunction in severe COVID-19 correlate with worse outcomes, mirroring the “immunoparalysis” observed in septic shock [5, 6].

PD-L1, a pivotal immune checkpoint molecule, downregulates immune responses by engaging programmed death-1 (PD-1) protein on T cells, leading to their functional exhaustion [7, 8]. In sepsis, increased PD-L1 expression is implicated in monocyte dysfunction, impaired cytokine production, and poor clinical outcomes [5]. Circulating or soluble PD-L1 (sPD-L1) has emerged as a potential biomarker of immune suppression in patients with critical illness. Preclinical study shows that blocking this pathway can improve survival [9]. These findings position PD-L1 as a mechanistic marker of immune dysfunction as well as a potential prognostic indicator for critical illness.

Recent evidence suggests that the PD-1/PD-L1 pathway is also dysregulated in severe COVID-19 cases [10, 11]. Beserra et al. reported significantly higher sPD-L1 concentrations in hospitalized patients with COVID-19 than in healthy controls, suggesting its role in the pathophysiology of severe infection [11]. In other studies, a “storm” of soluble immune checkpoints, including PD-L1, has been identified, which correlates with disease severity [12]. These data suggest that PD-L1 may contribute to COVID-19-associated immune dysregulation, consistent with observations in bacterial sepsis.

Currently, the lack of longitudinal data on PD-L1 dynamics during critical illness represents a significant gap in the literature. In most studies, PD-L1 (or sPD-L1) has been assessed at a single time point, such as upon hospital admission, providing a limited snapshot of the disease [9, 10]. The evolution of PD-L1 levels during intensive care remains unclear, and it is unknown whether these changes correlate with clinical outcomes. Dynamic monitoring of immune checkpoints, such as PD-1/PD-L1, has been proposed for prognosis and immunotherapy in sepsis [5]; however, longitudinal studies remain rare. Temporal data on PD-L1 are particularly scarce in COVID-19. One study reported no significant change in sPD-L1 levels over time after severe acute respiratory syndrome coronavirus 2 (SARS-CoV-2) infection, but only a few time points were analyzed [11]. Overall, it remains uncertain whether PD-L1 levels remain elevated, fluctuate, or decline during ICU stay. Understanding these patterns could help identify transitions between hyperinflammatory and immunosuppressive phases, guiding the optimal timing of immunomodulatory interventions.

To address this gap, our study was designed to characterize the longitudinal trajectory of PD-L1 levels in patients with critical illness owing to COVID-19 and evaluate the prognostic significance of these time-dependent changes. We hypothesized that patients who died would show persistently elevated or increasing PD-L1 levels, whereas survivors would show declining levels. By analyzing the temporal dynamics of PD-L1 levels in this study, we aimed to provide novel insights into the host immune response in severe COVID-19 and determine whether PD-L1 could serve as a dynamic prognostic biomarker in critical care.

## Methods

### Study design and participants

This single-center, retrospective observational study was conducted at Mie University Hospital between April 2021 and December 2022. We included 40 consecutive adult patients hospitalized with laboratory-confirmed COVID-19, defined by a positive reverse-transcription polymerase chain reaction assay for SARS-CoV-2, in the analysis. We also recruited 23 age- and sex-matched healthy volunteers to serve as the control group. Patients were excluded if key variables were missing or if informed consent could not be obtained. The study protocol was approved by the Institutional Review Board of Mie University Hospital (Approval numbers: IRB No. 3026 and H2021-191). We obtained written informed consent from the patients or their next of kin upon admission to the hospital.

### Data collection and measurements

Demographic data, comorbidities, and laboratory values were obtained from the electronic medical records. Plasma samples were collected within 24 h of hospital admission (ICU day 1) and subsequently on ICU day 5, 7, 14, and 21 of hospitalization. Plasma samples were obtained in ethylenediaminetetraacetic acid-containing tubes and stored at –80 °C until use. PD-L1 levels were measured using the Human PD-L1 SimpleStep Enzyme-Linked Immunosorbent Assay Kit (28–8 clone; Abcam, ab277712, Cambridge, UK) according to the manufacturer’s instructions. We also routinely performed blood tests, including complete blood counts, coagulation studies, and biochemical assays, to monitor the clinical status and severity of the disease. The primary outcome was in-hospital mortality.

### Statistical analysis

Continuous variables are expressed as mean ± standard deviation (SD) or median interquartile range (IQR) and were compared using Student’s t-test or Mann–Whitney U test. Categorical variables were analyzed with Fisher’s exact test.

Kaplan–Meier survival analysis, along with a log-rank test, was used to compare mortality between groups and assess statistical significance. The Cox proportional hazards regression model was used to identify predictors of mortality. Furthermore, it was implemented using the lifelines Python package to incorporate longitudinal data. All 46 candidate predictors were first analyzed using separate univariable Cox models. We subsequently adjusted *p*-values for multiplicity using the Benjamini–Hochberg procedure, controlling the false discovery rate (FDR) at 5%. Predictors with an FDR-adjusted q-value < 0.05, along with prespecified clinically essential covariates, were carried forward into the multivariable analysis.

To prevent overfitting in this small data set (10 events), we restricted the final model to three clinically relevant variables (event-per-variable ratio ≈ 3.3) and applied L1-penalized (LASSO) Cox regression. The penalization parameter (λ) was selected via grid search over 20 candidate values on a logarithmic scale (10⁻^4^–1.0), using stratified threefold cross-validation to maximize the mean concordance index; the cross-validated optimal λ (≈ 0.003) was then used to fit the final model. Model optimism was quantified using 1000 bootstrap resampling to obtain optimism-corrected C-indices and confidence intervals, and the stability of the three coefficients was evaluated as their selection frequency (non-zero coefficients) across bootstrap samples. Simulation studies suggest that under strong penalization, acceptable bias and calibration can be maintained with event-to-variable ratios as low as 5 [13, 14]. Therefore, the resulting model should be considered hypothesis-generating, and its stability will require confirmation in larger, independent cohorts.

### Machine learning models

Machine learning (ML) modeling was performed in Python 3.10 using scikit-learn v1.4 and Shapley Additive Explanations (SHAP) v0.45.0. The analysis included all admission-time laboratory and clinical variables, along with serial sPD-L1 concentrations (ICU day 1, 5, 7, 14, and 21). We retained variables with ≤ 25% missing data, which were replaced with the column mean. Non-informative identifiers and the binary outcome label were excluded from the predictor matrix. Continuous predictors were z-transformed using StandardScaler, with scaling parameters estimated on each training fold and subsequently applied to the corresponding test fold to prevent data leakage.

We evaluated eight supervised classifiers: support vector machine (SVM), neural network (NN), decision tree (DT), AdaBoost, gradient boosting machine (GBM), linear discriminant analysis (LDA), logistic regression, and random forest (RF). Hyperparameters for SVM, NN, DT, and RF were tuned using a grid search (Additional file 1).

Model development followed a stratified fivefold cross-validation scheme. Within each training fold, an inner grid search was conducted to identify the optimal hyperparameter set (scoring = area under the receiver operating characteristic [ROC] curve [AUC]). The best estimator was then refitted on the full training fold and evaluated on the held-out test fold. We used the predicted class probabilities to construct ROC curves and to calculate the AUC for each fold. True-positive rates were interpolated at 100 equally spaced false-positive rate points, and mean ROC curves were generated across folds for every algorithm.

Model interpretability was assessed using SHAP. KernelExplainer was fitted on the training data, and global feature importance was ranked based on mean absolute SHAP values. All computations were executed with a fixed random seed (random_state = 42). Given the small cohort (40 patients; 10 events), the ML component remains at risk of overfitting. Stratified fivefold cross-validation provides internal, not external, validation, so performance may be optimistically biased; therefore, these results should be regarded as exploratory/hypothesis-generating, pending external validation in a larger cohort.

### Software and tools

All statistical and ML analyses were performed using Python version 3.11. The following libraries were used: lifelines (for survival modeling) and scikit-learn (for ML model training). Data preprocessing and visualization were conducted using pandas, NumPy, Matplotlib, and Seaborn.

## Results

### Patient characteristics

A total of 40 patients with severe COVID-19 who were admitted to Mie University Hospital were included in the study. Among them, 10 (25%) died during hospitalization. Table 1 shows a summary of the baseline characteristics and laboratory data at admission, stratified by survival status. The mean age was 59.8 ± 14.3 years in survivors and 64.3 ± 14.4 years in non-survivors (*p* = 0.109).

**Table 1:** Baseline characteristics of patients with COVID-19

	Survivors	Non-survivors	*p*-value
	n = 30	n = 10	
Age, years	59.78 ± 14.32	64.29 ± 14.44	0.109
Sex (female/male)	8/22	1/9	0.404
Body mass index, kg/m^2^	30.00 ± 7.75	27.67 ± 5.44	0.739
SOFA score	3.57 ± 1.77	7.40 ± 3.60	< 0.001
Glasgow Coma Scale	13.40 ± 3.77	11.50 ± 5.02	0.396
P/F ratio	202.36 ± 112.84	175.01 ± 58.76	0.612
APACHE II score	13.40 ± 3.77	16.50 ± 7.20	0.018
Comorbidities			
Hypertension	14	5	1.000
Diabetes mellitus	11	5	0.482
Kidney disorder	1	3	0.042
Respiratory disorder	8	1	0.404
Cardiovascular disorder	4	3	0.338
Liver disorder	0	2	0.058
Immune disease	1	0	1.000
CBC			
Hemoglobin(g/dL)	13.41 ± 1.40	12.84 ± 2.93	0.656
WBC (× 10^3^/μL)	9.32 ± 5.01	7.75 ± 4.09	0.301
Lymphocyte (%)	9.42 ± 6.22	13.26 ± 10.76	0.48
Neutrophil (%)	84.98 ± 7.88	78.58 ± 13.54	0.18
Platelet (× 10^3^/μL)	249.10 ± 107.60	182.67 ± 102.74	0.036
Coag			
D-dimer (μg/mL)	5.31 ± 8.56	28.26 ± 74.65	0.301
PT-INR	1.17 ± 0.26	1.18 ± 0.25	0.818
APTT (s)	34.50 ± 5.73	35.94 ± 5.30	0.548
Fibrinogen (mg/dL)	522.97 ± 148.45	441.90 ± 170.84	0.155
Chem			
Total protein (g/dL)	6.51 ± 0.56	6.13 ± 0.56	0.109
Albumin (g/dL)	2.75 ± 0.50	2.60 ± 0.59	0.89
Creatinine (mg/dL)	0.90 ± 0.52	2.40 ± 1.80	0.009
BUN (mg/dL)	23.85 ± 17.62	45.47 ± 29.80	0.046
LDH (U/L)	536.23 ± 181.92	548.80 ± 435.44	0.379
CRP (mg/dL)	11.83 ± 6.51	8.33 ± 7.12	0.123
ABG			
pH	7.35 ± 0.10	7.28 ± 0.16	0.379
PaO_2_ (mmHg)	116.20 ± 47.61	110.39 ± 57.87	0.382
PaCO_2_ (mmHg)	44.32 ± 13.83	49.83 ± 19.40	0.59
Lactate (mmol/L)	1.55 ± 0.62	3.02 ± 3.29	0.023
Bicarbonate (mmol/L)	23.29 ± 3.52	22.06 ± 4.03	0.469
Treatments			
Corticosteroids	28	9	1.000
Remdesivir	23	4	0.054
Heparin	27	7	0.153
Tocilizumab	25	8	0.190

Values are presented as mean ± SD, median [IQR], or n (%), as appropriate. For between-group comparisons of survivors (n = 30) and non-survivors (n = 10), a two-tailed Student’s *t*-test was used for approximately normally distributed continuous variables or the Mann–Whitney *U* test otherwise; categorical variables were compared using Fisher’s exact test

The significance level was α = 0.05, with no adjustment for multiple comparisons

All measurements (including arterial blood gases and lactate) were obtained at ICU admission

Available cases were used for the analysis; variable-specific sample sizes may differ where data were missing

SOFA: Sequential Organ Failure Assessment; P/F ratio: partial pressure of arterial oxygen to fraction of inspired oxygen ratio; APACHE II: Acute Physiology and Chronic Health Evaluation II; CBC: complete blood count; WBC: white blood cell count; Coag: coagulation tests; PT-INR: prothrombin time—International Normalized Ratio; aPTT: activated partial thromboplastin time; Chem: blood chemistry; BUN: blood urea nitrogen; LDH: lactate dehydrogenase; CRP: C-reactive protein; ABG: arterial blood gas analysis; PaO₂: arterial oxygen pressure; PaCO₂: arterial carbon dioxide pressure; SD: standard deviation; IQR: interquartile range; ICU: intensive care unit

Non-survivors had significantly higher Sequential Organ Failure Assessment (SOFA) scores (7.40 ± 3.60 vs. 3.57 ± 1.77; *p* < 0.001) and lower platelet counts (182.7 ± 102.7 vs. 249.1 ± 107.6 × 10^3^/μL; *p* = 0.036) than did their counterparts. Creatinine and blood urea nitrogen (BUN) levels were also significantly elevated in the non-survivor group (creatinine: 2.40 ± 1.80 vs. 0.90 ± 0.52 mg/dL, *p* = 0.009; BUN: 45.5 ± 29.8 vs. 23.9 ± 17.6 mg/dL, *p* = 0.046), suggesting impaired renal function.

There were no significant differences in pharmacological treatments between survivors and non-survivors. The proportions of patients receiving corticosteroids, remdesivir, heparin, and tocilizumab were comparable between the two groups (*p* = 1.000, 0.054, 0.153, and 0.190, respectively).

Other variables, including Glasgow Coma Scale score, partial pressure of arterial oxygen to fraction of inspired oxygen ratio, inflammatory markers, coagulation parameters, and arterial blood gas values, showed no significant differences between groups.

### Plasma programmed death-ligand 1 levels

**Fig. 1:**
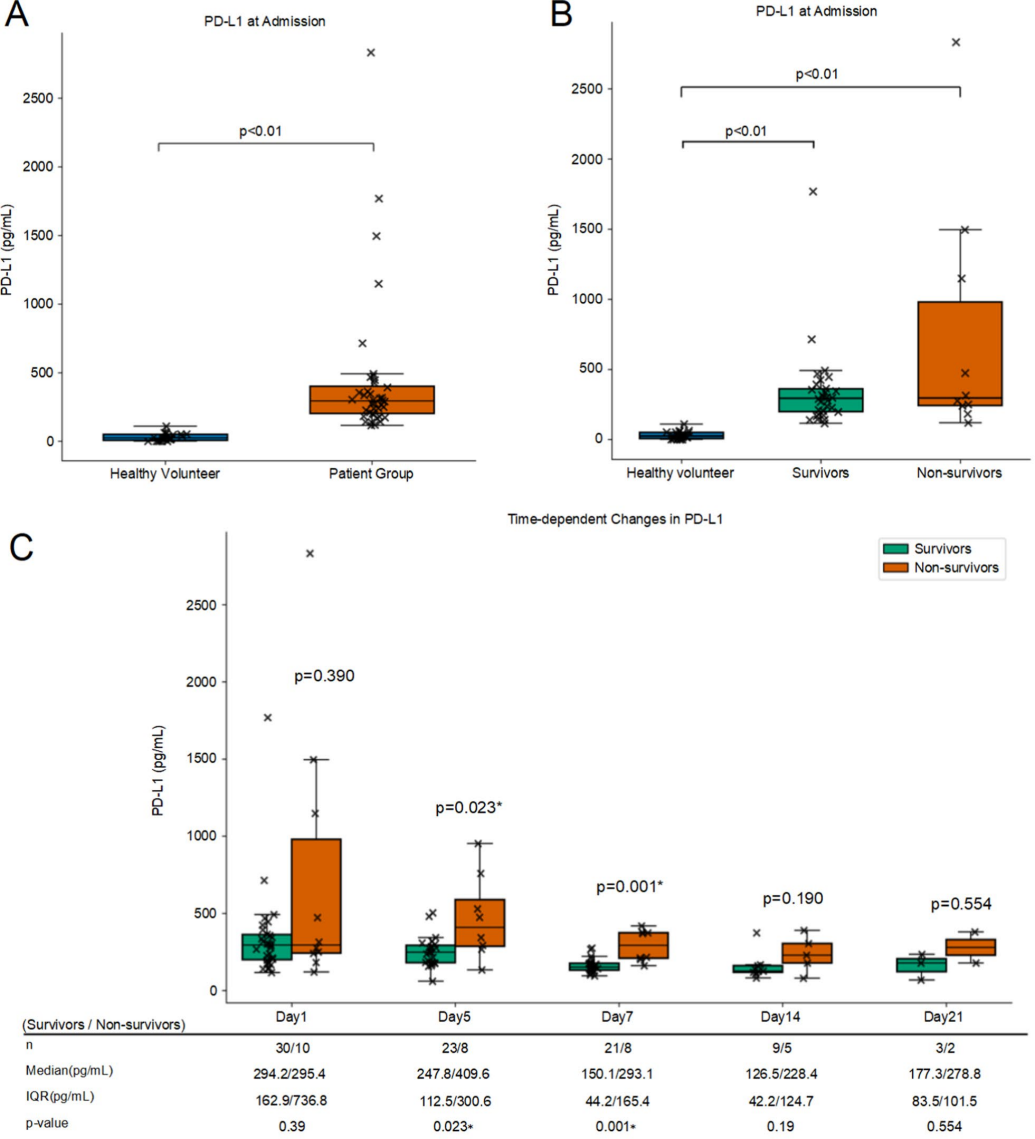
Plasma sPD-L1 at admission and longitudinally in COVID-19. **A** Comparison of plasma PD-L1 levels at admission between healthy volunteers and the overall patient group. **B** Plasma PD-L1 levels at admission in healthy volunteers, COVID-19 survivors, and non-survivors. **C** Longitudinal sPD-L1 in survivors vs. non-survivors on ICU day 1, 5, 7, 14, and 21 after admission; group sizes (survivors/non-survivors): day 1, 30/10; day 5, 23/8; day 7, 21/8; day 14, 9/5; and day 21, 3/2. Boxes show interquartile range (IQR); center line represents the median; whiskers indicate 1.5 × IQR; points denote individuals. Two-tailed Mann–Whitney U tests were used for between-group comparisons at each time point; *p* < 0.05 is considered statistically significant (asterisk indicates *p* < 0.05). PD-L1, programmed death-ligand 1; IQR, interquartile range; COVID-19, coronavirus disease 2019; sPD-L1, soluble programmed death-ligand 1

Plasma PD-L1 levels were significantly higher in patients with COVID-19 than in healthy controls at admission (median [IQR]: 294.15 vs. 55.88 pg/mL, *p* < 0.001). However, no significant difference was observed between survivors and non-survivors at admission (*p* = 0.390) (Fig. 1A, B).

Longitudinal analysis revealed a decreasing trend in PD-L1 levels over time in both groups. Notably, PD-L1 concentrations remained higher in non-survivors throughout the observation period. Significant differences were detected on ICU day 5 and 7 (*p* = 0.023 and *p* = 0.001, respectively), but not on ICU day 1, 14, and 21 (Fig. 1C).

### Correlation between programmed death-ligand 1 levels and clinical parameters

**Fig. 2:**
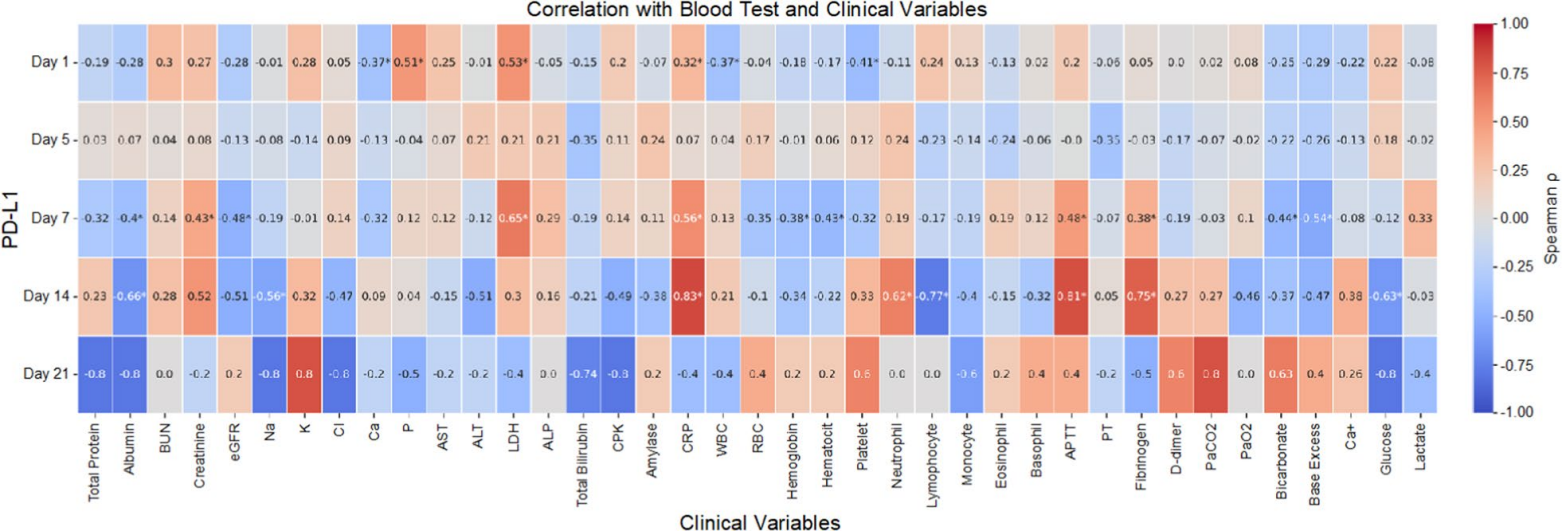
Spearman correlations between plasma sPD-L1 levels and clinical variables in patients with severe COVID-19. The heatmap shows Spearman’s rank correlation coefficients (ρ) between sPD-L1 concentrations and selected clinical parameters measured on intensive care unit days 1, 5, 7, 14, and 21. Warmer colors indicate positive correlations, and cooler colors represent negative correlations. Numerical ρ values are displayed in each cell. Asterisks (*) indicate significant correlations after two-tailed testing (*p* < 0.05). Analyses used available samples at each time point. eGFR, estimated glomerular filtration rate; AST, aspartate aminotransferase; ALT, alanine aminotransferase; ALP, alkaline phosphatase; CPK, creatine phosphokinase; RBC, red blood cell count; sPD-L1, soluble programmed death-ligand 1

We performed a Spearman correlation analysis to explore the relationship between PD-L1 levels and various clinical and laboratory parameters at five time points (ICU day 1, 5, 7, 14, and 21). The results are summarized as a heatmap in Fig. 2, with correlation coefficients annotated in each cell. Statistically significant correlations (*p* < 0.05) are marked with an asterisk (*).

A statistically significant inverse correlation was observed between platelet count and sPD-L1 level at admission (ICU day 1), indicating that higher PD-L1 concentrations were associated with lower platelet counts from the outset of critical illness. Conversely, creatinine showed a positive correlation with sPD-L1 level from ICU day 1 through day 14, becoming statistically significant on ICU day 7.

### Cox proportional hazards analysis

Forty-six longitudinal candidate variables were each entered into separate Cox models. After Benjamini–Hochberg adjustment (FDR = 5%), six predictors met the prespecified threshold (q < 0.05) and were therefore eligible for multivariable modeling. The strongest signals were ICU day 7 sPD-L1 (per-SD hazard ratio [HR] = 2.09, 95% confidence interval [CI] 1.27–3.42, q = 0.037) and arterial lactate at admission (per-SD HR = 2.69, 95% CI 1.42–5.10, q = 0.037). Additional predictors passing FDR control were creatinine (per-SD HR = 2.10, q = 0.037), total bilirubin (per-SD HR = 2.20, q = 0.044), amylase (per-SD HR = 1.98, q = 0.037), and SOFA score (per-SD HR = 2.10, q = 0.037). The corresponding SDs for each predictor, along with the complete screening statistics, are provided in Additional file 2.

Subsequently, we applied LASSO-Cox regression, limited to three clinically plausible covariates: lactate, sPD-L1 (ICU day 7), and creatinine. The penalization parameter (λ) was selected via a stratified threefold cross-validated grid search (optimal λ ≈ 0.003). The final coefficients and HRs are summarized in Table 2. This penalized model achieved a concordance (Harrell’s C-index) of 0.90, with a partial Akaike information criterion of 45.4, indicating good apparent discrimination. Using 1,000 bootstrap resamples, the optimism estimate was 0.043, yielding an optimism-corrected C-index of 0.853. After shrinkage, higher lactate and elevated ICU day 7 sPD-L1 remained independently associated with an increased instantaneous risk of death (approximately 4.4-fold and 4.0-fold, respectively). Plasma creatinine was not statistically significant post-penalization.

**Table 2:** Penalized multivariable Cox model for in-hospital mortality

	β (log-HR)	HR	95% CI	*p*-value (q)
Lactate	1.49	4.42	1.79–10.92	< 0.005 (0.009)
ICU day 7 sPD-L1	1.38	3.98	1.47–10.76	0.010 (0.028)
Creatinine	− 0.28	0.76	0.38–1.51	0.43 (0.61)

Values include multivariable penalized Cox proportional hazards regression analysis of arterial lactate, ICU day 7 plasma sPD-L1, and plasma creatinine levels

Data shown are the regression coefficients β (log-HRs), corresponding HRs, 95% CIs, and two-sided *p*-values (Wald tests); q-values are *p*-values adjusted for multiple testing (false discovery rate)

For continuous predictors, HRs represent the relative change in hazard per 1-unit increase in the predictor

CI: confidence interval; HR: hazard ratio; sPD-L1, soluble programmed death-ligand 1

### Risk stratification based on soluble programmed death-ligand 1 and lactate

**Fig. 3:**
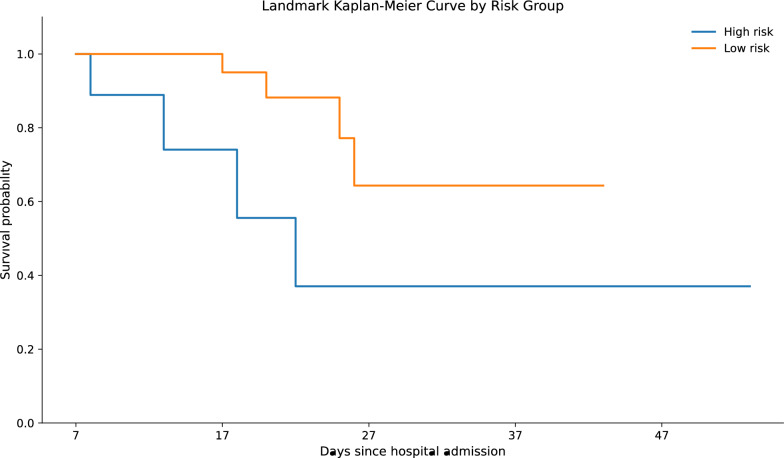
Kaplan–Meier survival curves stratified by combined the ICU day 7 sPD-L1 and arterial lactate risk groups in severe COVID-19. Survival probability is shown for the high-risk (orange) and low-risk (blue) cohorts identified in the study population (N = 40). Patients were classified as high-risk when plasma sPD-L1 on intensive care unit (ICU) day 7 exceeded the median (171 pg mL⁻^1^) and their arterial lactate exceeded the median (1.4 mmol L⁻^1^). All others were categorized as low risk. Censored observations are marked by vertical ticks. The survival difference between groups was significant using the log-rank test (χ.^2^ = 4.42, df = 1, *p* = 0.036). sPD-L1, soluble programmed death-ligand 1; COVID-19, coronavirus disease 2019

Patients were dichotomized at a prespecified ICU day 7 landmark using the median cut-offs of soluble PD-L1 on ICU day 7 (171 pg mL⁻^1^) and arterial lactate at admission (1.4 mmol L⁻^1^). Individuals exceeding both thresholds were classified as high-risk; all others formed the low-risk group. Before the ICU day 7 landmark, two deaths and one censoring occurred and were excluded from the risk set; thus, the ICU day 7 population comprised nine high-risk and 28 low-risk patients. Kaplan–Meier curves were plotted from ICU day 7 onward (x-axis displayed as days since admission, starting at ICU day 7) and showed a significant separation between strata (log-rank χ^2^ = 4.42, df = 1, *p* = 0.036) (Fig. 3).

### Machine learning-based mortality prediction

**Fig. 4:**
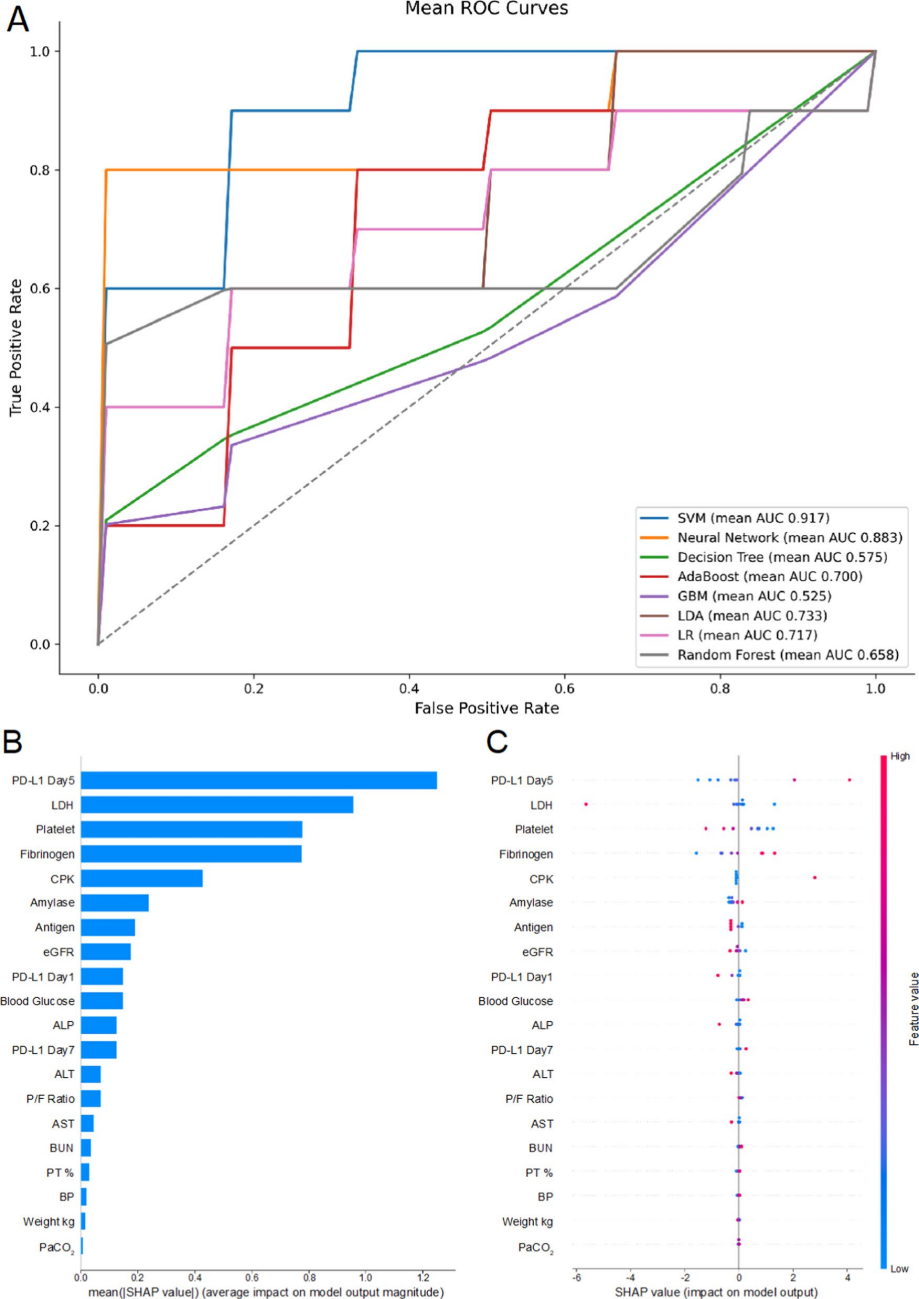
Machine learning performance and SHAP attribution for mortality. **A** ROC curves generated using stratified fivefold cross-validation for SVM, NN, DT, AdaBoost, GBM, LDA, LR, and RF. The mean AUC-ROC for each model is displayed in the inset; SVM achieved the highest discrimination (AUC = 0.917). The diagonal dashed line indicates no discrimination. **B** Global feature importance ranked by the mean absolute SHAP values for the best SVM classifier. ICU day 5 sPD-L1 was the most significant predictor, followed by lactate dehydrogenase, platelet count, and fibrinogen. **C** Beeswarm SHAP plot illustrating the direction and magnitude of impact of each feature on the SVM mortality prediction for individual patients. Positive SHAP values (rightward shift) increase the risk of death, whereas negative values (leftward shift) decrease it. Continuous variables are color-coded from low (blue) to high (red) values. AUC, area under the curve; CRP, C-reactive protein; DT, decision tree; FiO₂, fraction of inspired oxygen; GBM, gradient boosting machine; LDA, linear discriminant analysis; LDH, lactate dehydrogenase; LR, logistic regression; NN, neural network; P/F, PaO₂/FiO₂ ratio; RF, random forest; ROC, receiver operating characteristic; SHAP, SHapley Additive Explanation; sPD-L1, soluble programmed death-ligand 1; SVM, support vector machine

To further explore the predictive potential of clinical variables, including PD-L1 level, we trained and evaluated eight classification algorithms to predict in-hospital mortality. Model performance was assessed using stratified fivefold cross-validation, and mean AUC values were reported. SVM showed the highest performance (mean AUC = 0.917), followed by NN (AUC = 0.883) and LDA (AUC = 0.733). In contrast, the DT and GBM showed lower predictive accuracy (Fig. 4A).

SHAP analysis, conducted using the SVM model, revealed that PD-L1 levels on ICU day 5 were the most significant predictor of mortality, followed by lactate dehydrogenase, platelet count, and fibrinogen levels (Fig. 4B). The beeswarm plot (Fig. 4C) showed that higher PD-L1 levels were associated with higher predicted mortality, whereas higher platelet and fibrinogen levels were associated with decreased risk.

## Discussion

In our cohort of patients with COVID-19 who were critically ill, circulating sPD-L1 generally declined during the first 3 weeks of hospitalization, yet remained persistently elevated in non-survivors. sPD-L1 measurements taken on ICU day 5 and 7 distinguished survivors from non-survivors, indicating that sustained sPD-L1 elevation was associated with adverse outcomes. ICU Day 7 sPD-L1 and admission lactate levels were further identified via multivariable Cox analysis as independent predictors of in-hospital mortality; the prognostic value of lactate aligns with previous reports [15–17]. Taken together, these observations indicate that higher sPD-L1 levels, elevated lactate, immune dysregulation, and tissue hypoxia may help identify patients at increased risk of in-hospital death.

As shown in Fig. 2, higher sPD-L1 levels were associated with earlier renal dysfunction. Likewise, inflammatory and coagulation indices, such as C-reactive protein (CRP) and fibrinogen, both showed a positive correlation with sPD-L1 levels on multiple ICU days. These correlations suggest that sPD-L1 may reflect a composite burden of renal dysfunction, systemic inflammation, and coagulation activation during critical illness, potentially reflecting elements of a shared pathophysiological axis. Accordingly, sPD-L1 warrants consideration as a complementary biomarker alongside established prognostic indices.

In moderate-to-severe COVID-19, immunologic heterogeneity is pronounced, with some patients exhibiting marked inflammatory responses while others show prolonged viral replication (such as RNAemia) [18]. The latter phenotype has been linked to a higher risk of multiorgan failure and death [19], whereas cytokine storm is not uniformly present across severe cases [20]. In our cohort, the sPD-L1 levels at admission were high and widely dispersed, consistent with a mixture of inflammatory and persistent-infection states. By ICU day 5–7, group separation became more apparent, and sPD-L1 levels differed between outcome groups. Persistent sPD-L1 elevation may reflect ongoing viral replication, hypoxia, and metabolic stress, and measurements at ICU day 5–7 may more sensitively capture this immunologic phase transition.

Therapeutically, the benefit of immunomodulation is phase-dependent. Corticosteroids can be useful in hypoxemic severe disease, yet may be harmful in patients with protracted viral replication; indiscriminate immunosuppression should therefore be avoided [18, 21]. The poorer outcomes observed in patients receiving baseline corticosteroids for autoimmune disease further underscore the potential downsides of inappropriate immune suppression [22]. From this perspective, serial sPD-L1 monitoring could support clinical stratification and the timing of immune interventions, though interpretation should not be focused on a single biomarker and must remain cautious.

Prior studies have reported upregulated PD-L1 in lung tissue and elevated circulating sPD-L1 at a single time point in COVID-19 and other infections [11, 23]. Our findings build on the previous studies by profiling sPD-L1 longitudinally and showing that ICU day 5–7 values were more prognostically discriminative than were admission levels, thereby extending existing evidence.

Complementary evidence was obtained from the SVM model, which incorporated ICU day 1 and 7 sPD-L1 levels, along with other routine variables, including age, SOFA scores, and CRP levels. This model achieved excellent discrimination (AUC > 0.91), and based on SHAP attribution, the ICU day 7 sPD-L1 levels were ranked as the dominant predictor of mortality. The difference in importance ranking between the Cox and SVM models likely reflects variance inflation because of multicollinearity, as well as the capacity of non-linear kernels to capture complex interactions that linear proportional hazards models cannot [24, 25]. Overall, our findings support prior studies reporting elevated sPD-L1 in severe COVID-19 cases [11, 26]. Critically, we expand on these observations by demonstrating that the temporal trajectory of sPD-L1 is directly tied to patient prognosis, as evaluated using both classical regression and modern ML methods.

By design, the multivariate Cox regression and ML analysis in this study are exploratory in a small cohort and thus subject to overfitting and limited generalizability. Nevertheless, the focus on the PD-1/PD-L1 axis offers novelty beyond conventional prognostic indices, and our observation that persistent elevation of sPD-L1 is associated with adverse outcomes supports the clinical relevance of immune paralysis. Our analysis does not establish superiority of sPD-L1 over conventional organ-dysfunction or inflammation-based scores. Rather, its value appears to lie in complementing existing indices by adding an immunologic dimension to risk assessment.

The PD-1/PD-L1 axis acts as an immune checkpoint that helps dampen the activation of PD-1-expressing T-cells during inflammatory stress [7]. In COVID-19, as sPD-1 retains the ability to signal through PD-1 and suppress activated T cells, sPD-L1 may facilitate viral immune evasion during the early infection phase, ultimately dampening an overexuberant host response [23]. Therefore, the sustained elevation of sPD-L1 levels observed on ICU day 7 likely signals a shift toward the late, immunosuppressive phase of the disease, characterized by T cell exhaustion. This persistent PD-L1 expression may be driven by a late surge of interferon-γ, interleukin-6, and hypoxia-inducible factors (HIFs), which upregulate PD-L1 through signal transducer and activator of transcription 1 (STAT1)/interferon regulatory factor 1 (IRF1) or HIF-1α pathways [21, 27]. Such cytokine- and hypoxia-dependent regulation is consistent with the positive correlation that we observed between ICU day 7 sPD-L1 and the levels of lactate and CRP, with lactate serving as a marker of tissue hypoxia and CRP as an indicator of systemic inflammation.

A similar phenomenon is well documented in bacterial sepsis, where persistent PD-1/PD-L1 signaling drives prolonged immune paralysis [28], and PD-L1-deficient mice exhibit improved survival [29]. PD-1/PD-L1 blockade has yielded favorable outcomes in preclinical sepsis models; however, its therapeutic benefit in clinical trials has yet to be demonstrated [9, 28]. Our observational study provides pathophysiological evidence that sustained PD-L1 upregulation occurs during critical illness because of viral infection. These findings suggest that if checkpoint inhibition is used therapeutically, the timing of administration may be crucial to clinical efficacy.

sPD-L1 shows promise as a clinically useful prognostic marker. Rapid assessment on ICU admission and again on ICU day 7 could enable early identification of patients with COVID-19 who are critically ill and at heightened risk of death, thereby allowing for timely escalation or modulation of immunotherapeutic strategies. In our study, adding sPD-L1 levels to an ML model based on routine blood tests improved its predictive accuracy, highlighting its added value in multivariable risk scoring. Expanding this approach to include other soluble immune checkpoint molecules such as sCD40, soluble T-cell immunoglobulin and mucin domain (TIM) 1, and galectin-9, which have also been linked to disease severity [26, 30], could facilitate more granular immune profiling and guide personalized treatment algorithms in future studies.

This study has some limitations. First, it was a small, single-center observational cohort; therefore, the generalizability of our findings and any inference regarding therapeutic impact are restricted. Second, our analyses were focused solely on sPD-L1. A more comprehensive appraisal of host immune dysfunction would require simultaneous quantification of additional immune checkpoint mediators, such as PD-1, TIM-3, and lymphocyte-activation gene 3, coupled with functional T-cell assays.

Third, all multivariable and ML analyses are exploratory and at risk of overfitting given the limited sample and event counts. While we applied stratified cross-validation and penalization, cross-validation constitutes internal rather than external validation; performance estimates in small datasets may therefore be optimistically biased, and confirmation in independent cohorts is essential before any clinical use. Fourth, concomitant treatments (including corticosteroids, remdesivir, anticoagulants, and tocilizumab) varied across patients. These are summarized in Table 1; however, the study was underpowered for adjusted modeling of treatment effects, and unmeasured confounding cannot be excluded. Accordingly, all ML and treatment-related findings should be considered hypothesis-generating.

Finally, because randomized trials have not yet shown unequivocal benefit from PD-1/PD-L1 blockade in sepsis [31, 32], the clinical utility of targeting this axis in severe COVID-19 remains speculative and requires rigorous testing. Validation in larger, multicenter, longitudinal cohorts, together with broad-spectrum immune profiling and explicit modeling of time-varying treatments, is required to confirm our observations and determine their translational relevance.

## Conclusions

In this study, we found that persistently elevated plasma sPD-L1 levels are associated with poor prognosis in patients with COVID-19 who were critically ill. This suggests that sPD-L1–mediated immune suppression could contribute to disease progression and mortality in severe cases.

sPD-L1 and lactate levels were used for risk stratification to effectively predict in-hospital mortality. ML models further demonstrated the clinical utility of these markers. sPD-L1 may serve as both a prognostic biomarker and a potential therapeutic target, and further studies are warranted to validate its role in the immunopathogenesis of COVID-19.

## Supplementary Information

Additional file 1.

Additional file 2.

## Data Availability

Data will be shared upon reasonable request and institutional approval.

## Abbreviations

AUC: Area under the curve
CI: Confidence interval
COVID-19: Coronavirus disease 2019
CRP: C-reactive protein
DT: Decision tree
GBM: Gradient boosting machine
HR: Hazard ratio
ICU: Intensive care unit
IQR: Interquartile range
LDA: Linear discriminant analysis
ML: Machine learning
NN: Neural network
PD-1: Programmed death-1
PD-L1: Programmed death-ligand 1
RF: Random forest
ROC: Receiver operating characteristic
SARS-CoV-2: Severe acute respiratory syndrome Coronavirus 2
SHAP: SHapley additive explanations
SOFA: Sequential Organ Failure Assessment
sPD-L1: Soluble programmed death-ligand 1
SVM: Support vector machine
TIM: T-cell immunoglobulin and mucin
